# Supplementary material for: Ovariectomy uncouples lifespan from metabolic health and reveals a sex-hormone-dependent role of hepatic mTORC2 in aging
Source: eLife. 2020 Jul 28;9:e56177. doi: 10.7554/eLife.56177 (PMC7386906; doi:10.7554/eLife.56177)
Supplement: Transparent reporting form [file elife-56177-transrepform.docx]

***eLife’s* transparent reporting form**

We encourage authors to provide detailed information *within their submission* to facilitate the interpretation and replication of experiments. Authors can upload supporting documentation to indicate the use of appropriate reporting guidelines for health-related research (see [EQUATOR Network](http://www.equator-network.org/%20)), life science research (see the [BioSharing Information Resource](https://biosharing.org/)), or the [ARRIVE guidelines](http://www.plosbiology.org/article/info:doi/10.1371/journal.pbio.1000412) for reporting work involving animal research. Where applicable, authors should refer to any relevant reporting standards documents in this form.

If you have any questions, please consult our Journal Policies and/or contact us: [editorial@elifesciences.org](mailto:editorial@elifesciences.org).

**Sample-size estimation**

- You should state whether an appropriate sample size was computed when the study was being designed
- You should state the statistical method of sample size computation and any required assumptions
- If no explicit power analysis was used, you should describe how you decided what sample (replicate) size (number) to use

Please outline where this information can be found within the submission (e.g., sections or figure legends), or explain why this information doesn’t apply to your submission:

The number of animals required for the lifespan study was initially calculated to be 20-30 per group based on the 30% reduction in male L-RKO lifespan we previously observed (Lamming *et. al*, 2014, *Aging Cell*), and then allowing for the possibility that castration or ovariectomy would only partially rescue the animals, leading to the need to detect an approximately 15% change in lifespan. We selected a target range as mouse breeding of genetically engineered mice is never precisely mendelian, and we then needed to perform surgery. This number of animals would provide 84-95% power to detect a 15% change in lifespan (Liang *et al.*, 2003, Experimental Gerontology). We achieved the target group size for all groups. Group sizes for *in vivo* metabolic assays were chosen as described (Bellantuono *et al*. 2020, *Nature Protocols*)

**Replicates**

- You should report how often each experiment was performed
- You should include a definition of biological versus technical replication
- The data obtained should be provided and sufficient information should be provided to indicate the number of independent biological and/or technical replicates
- If you encountered any outliers, you should describe how these were handled
- Criteria for exclusion/inclusion of data should be clearly stated
- High-throughput sequence data should be uploaded before submission, with a private link for reviewers provided (these are available from both GEO and ArrayExpress)

Please outline where this information can be found within the submission (e.g., sections or figure legends), or explain why this information doesn’t apply to your submission:

All experiments were performed once per age as indicated in the figures and figure legends, and all replicate information reference to biological replicates. Figure legends and supplemental tables contain full information regarding the number of independent biological replicates. Reasons for censoring of lifespan data are included in supplemental tables for each animal; no lifespan data for animals that successfully recovered from gonadectomy/sham surgery was excluded as described in Methods. No sequence data is included in this manuscript.

**Statistical reporting**

- Statistical analysis methods should be described and justified
- Raw data should be presented in figures whenever informative to do so (typically when N per group is less than 10)
- For each experiment, you should identify the statistical tests used, exact values of N, definitions of center, methods of multiple test correction, and dispersion and precision measures (e.g., mean, median, SD, SEM, confidence intervals; and, for the major substantive results, a measure of effect size (e.g., Pearson's r, Cohen's d)
- Report exact p-values wherever possible alongside the summary statistics and 95% confidence intervals. These should be reported for all key questions and not only when the p-value is less than 0.05.

Please outline where this information can be found within the submission (e.g., sections or figure legends), or explain why this information doesn’t apply to your submission:

Statistical analysis methods are fully described in the Figure Legends and Methods section, and Raw data is provided for all lifespan graphs in the provided supplemental tables and presented in the figures where informative to do so. Statistical tests for each experiment and number of animals per group are fully described in the figures and figure legends, and exact p-values for statistically significant results (p<0.05) are provided in the figures wherever possible.

(For large datasets, or papers with a very large number of statistical tests, you may upload a single table file with tests, Ns, etc., with reference to sections in the manuscript.)

**Group allocation**

- Indicate how samples were allocated into experimental groups (in the case of clinical studies, please specify allocation to treatment method); if randomization was used, please also state if restricted randomization was applied
- Indicate if masking was used during group allocation, data collection and/or data analysis

Please outline where this information can be found within the submission (e.g., sections or figure legends), or explain why this information doesn’t apply to your submission:

Animals were randomly assigned to sham or surgical groups following genotyping. No masking was used during group allocation, data collection, or data analysis, although experimenters were generally not aware of group assignment during the collection of in vivo metabolic data.

**Additional data files (“source data”)**

- We encourage you to upload relevant additional data files, such as numerical data that are represented as a graph in a figure, or as a summary table
- Where provided, these should be in the most useful format, and they can be uploaded as “Source data” files linked to a main figure or table
- Include model definition files including the full list of parameters used
- Include code used for data analysis (e.g., R, MatLab)
- Avoid stating that data files are “available upon request”

Please indicate the figures or tables for which source data files have been provided:

Source data files have been provided as raw tables S1-S4 for Figures 1 and 2.
